# From clinic to couch: a pilot study of home-use photobiomodulation for radiation-induced oral mucositis and dermatitis

**DOI:** 10.1007/s00520-025-10037-3

**Published:** 2025-11-07

**Authors:** Saeed Salman, Ragda Abdalla-Aslan, Ahmad Awawdi, Rana Tarabeih, Salem Billan

**Affiliations:** 1 Fishman Oncology Center, Rambam Health Care Campus, Haifa, Israel; 2 Oral and Maxillofacial Surgery Department, Rambam Health Care Campus, Haifa, Israel; 3 Ruth & Bruce Rappaport Faculty of Medicine, Technion-Israel Institute of Technology, Haifa, Israel

**Keywords:** Head and neck neoplasms, Intensity-modulated radiotherapy, Oral mucositis, Radiation-induced dermatitis, Photobiomodulation therapy, Low-level laser therapy

## Abstract

**Purpose:**

Radiation-induced oral mucositis (OM) and dermatitis (RD) are common debilitating toxicities of radiotherapy (RT) for head and neck cancer (HNC) patients. While photobiomodulation (PBM) has shown promise in reducing severity of these adverse effects, in-clinic treatments pose logistic and financial challenges. This study assessed the feasibility and compliance of a home-use, self-applied near-infrared PBM device for the prevention and treatment of RT-induced OM and RD.

**Methods:**

This prospective, single-arm trial enrolled 20 HNC patients scheduled for intensity-modulated RT (IMRT) with or without concurrent chemotherapy. Seventeen patients were analyzed following exclusions. Participants self-administered PBM at home twice daily for 9–11 weeks, from RT initiation to four weeks post-RT. The primary outcome was compliance (≥ 50% of expected treatments). Secondary outcomes included incidence of severe (grade 3–4) OM and RD.

**Results:**

67% of the participants completed at least 50% of the prescribed PBM treatments. Overall, 60% of expected sessions were administered, surpassing the predefined compliance threshold. By week 3, 6% presented with severe OM and none with severe RD. At RT completion, 41% and 18% had severe OM and RD, respectively. Four weeks post-RT, no severe OM or RD were observed, with the majority presenting with no toxicity (grade 0). No device related adverse effects were reported, and patients reported high comfort and ease of use.

**Conclusion:**

Self-administered, home-use PBM is a feasible and well-tolerated approach that shows promise as pre-emptive treatment for RT induced OM and RD. Large-scale, randomized, placebo-controlled trials are needed to confirm these findings and evaluate long-term benefits.

Trial registration: ClinicalTrials.gov Identifier: NCT05176834. Registration Date: December 13th, 2021.

## Background

Radiation induced oral mucositis (OM) and dermatitis are severe and immediate adverse effects of radiotherapy (RT) with or without chemotherapy for patients with head and neck cancer (HNC). Depending on the treatment protocol, these patients suffer from OM in 59.4% [1] to 100% of cases [2–7], with severe OM in 34%-85% [8, 9]. Radiation dermatitis (RD) affects up to 80–90% of HNC patients, with severe grade RD accounting for 30–57% of cases [10, 11].

These debilitating sequelae cause significant pain, loss of function, risk of infection, need for nutritional support and a decrease in quality of life [12, 13]. This can lead to a decrease or interruption of the therapeutic protocol, which may have negative prognostic implications. OM may raise medical costs due to the need for extensive supportive care and prolonged hospitalization [14, 15]. As a result, substantial research has been performed to uncover effective preventive and therapeutic measures for OM.

Photobiomodulation (PBM), previously known as low-level laser therapy, is the application of non-ionizing visible or near infrared light to tissue. Endogenous chromophores trigger photophysical and photochemical reactions in multiple biological pathways through a non-thermal method [16]. PBM promotes wound healing, reduced pain and offers anti-inflammatory properties [16].

PBM has been shown to have a positive effect on RT induced OM, and international guidelines for the use of PBM for prevention of OM were published by the UK National Institute for Health and Care Excellence (NICE) (IPG615) and by the Multinational Association of Supportive Care in Cancer, and the International Society of Oral Oncology (MASCC/ISOO) in adult patients receiving RT [17], using specific PBM parameters [18]. Furthermore, the World Association for Photobiomodulation Therapy (WALT) has issued recommendations supporting PBM for the treatment of established OM [19].

Similarly, PBM had been shown to be effective in preventing severe RD in breast cancer and possibly in HNC patients [20–22]. These systematic reviews highlight the need for further large-scale randomized trials and standardization of treatment protocols before PBM can be universally recommended as standard of care. Based on these and a Delphi consensus [23], MASCC recommended PBM use for the management of RD in patients with breast cancer [24]. A recent international multidisciplinary expert consensus concluded that PBM is a safe and effective treatment option for acute RD in adults [25]. Similarly, recent practical recommendations by European Society for Radiotherapy and Oncology (ESTRO) Radiation Therapist (RTT) Committee suggested considering PBM for prevention of RD [26].

However, the current evidence came from research conducted on office-based PBM devices administered by professional health care providers, which necessitates several frequent clinic appointments. According to a study that evaluated the real-life experiences of MASCC/ISOO mucositis study group members, the biggest challenges to adopting office PBM include financial constraints, time, lack of experience, country-specific legislation, and patient refusal [27]. In addition, a recent study emphasized the challenges from the patient's perspective. HNC patients who received PBM twice weekly over a 6-week period noted difficulties due to traffic, travel duration, and distance from the study location [28]. Furthermore, repeated clinic visits may constitute a major health risk for these immunocompromised patients. In addition to offering a solution to these challenges, self-treatment using a home device has also the advantage of empowering the patient.

This prospective, single-arm clinical trial was designed to evaluate the feasibility and potential efficacy of home-use self-applied near-infrared PBM device (B-Cure Laser Pro, Erika B-Cure, Haifa, Israel) for prevention and treatment of RT induced OM and RD in HNC patients. The device that has been certified for use in Israel, Canada, Europe, and Brazil and has shown to be effective in double blinded randomized controlled studies for treatment of temporomandibular joint pain [29] and diabetic foot ulcers [30]. Additionally, it has shown promising results in case series involving cancer-related OM, non-healing diabetic wounds, and drug-related dermatitis [31, 32].

This study serves as a preparatory step toward a larger, double-blind, randomized, sham-controlled trial aimed at determining the device’s potential to reduce the incidence and severity of these detrimental side effects.

## Methods

### Study design

This was a single center, prospective, single arm study. Patients with HNC, scheduled to undergo RT received a PBM home use personal device. Patients were required to self-treat twice a day during RT and up to 4 weeks later. Evaluations visits were carried at 4 time points: (1) baseline; (2) three weeks after RT initiation (expected time for OM occurrence); (3) end of the RT; (4) four weeks after the end of the RT or at complete healing of OM and RD (the earlier of the two). Evaluations included OM and RD severity assessed by the study team (SS and RAA) and patient reported outcomes including comfort and ease of device use, and Functional Assessment of Cancer Therapy-Head and Neck Symptom Index (FHNSI) [33]. The primary outcome was patient adherence to the self-treatment protocol based on home diaries. Secondary outcomes included incidence of severe (grade 3–4) OM and RD. Side effects were monitored throughout the study.

### Ethics

The study was approved by the institutional review board (0183–21-RMB), and prospectively registered (ClinicalTrials.gov NCT05176834). All patients signed an informed consent form before participating in the study.

### Study Population

The recruitment of volunteer participants was carried out from February 08 2022 until February 01, 2023. Inclusion criteria included adult patients over 18 years-old; both genders; HNC in the oral cavity, oropharynx, salivary glands or skin; patients who are scheduled for receiving intensity modulated RT (IMRT) for a period of 6–7 weeks, 5–6 fractions per week (a total of 60-70Gy) with or without concurrent chemotherapy and/or immunotherapy; the treatment field including at least one oral site (buccal mucosa, floor of the mouth, tongue, hard palate, retro-molar trigon, lips or oropharynx); an independent functional state (Performance Status Scale ECOG ≤ 2); the patient is able to undergo treatment intra-oral (e.g. no trismus); and signed informed consent.

Exclusion criteria included previous RT for the head and neck; the patients is unable to open their mouth; the patient receives medications intended for the treatment and/or prevention of OM that is not basic oral care, such as benzydamine mouthwash, morphine mouthwash, etc.; the patient is enrolled in any other clinical trial that includes treatment or intervention that can affect the course of OM; a medical background that affects wound healing (e.g. diabetes, lupus, etc.); and pregnancy.

### Treatment protocol

**Fig. 1:**
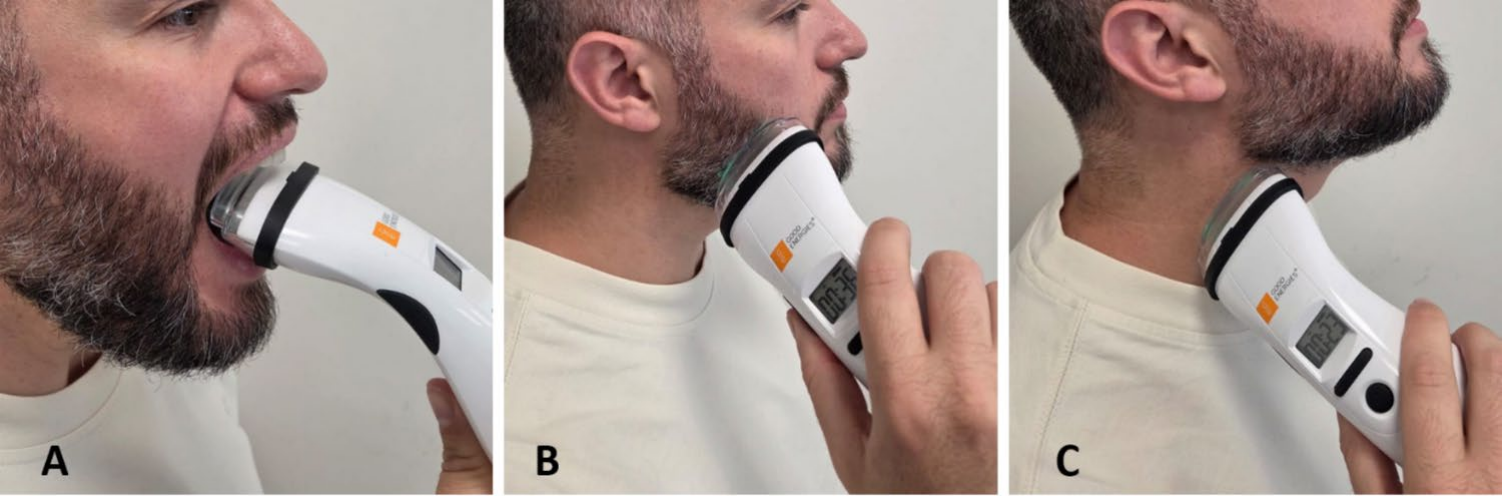
Protocol defined treatment sites: intra-oral (**A**), bilateral cheek area (**B**) and bilateral neck (**C**)

The treatment protocol was designed based on the literature and our experience, taking into account the device specifications [31]. Each participant received a B-Cure Pro (Erika B-Cure, Haifa, Israel) laser device for personal use at home, detailed instructions for use from the research team, and a diary to document the frequency of treatments. The energy parameters of the device are 808 nm wavelength, 250 mW peak power (55 mW/cm^2^), 15kHz, 1.1 J/cm^2^/min, while the ray size is 4.5 × 1.0cm^2^. The probe was applied in a stationary (spot) mode, targeting predefined mucosal and cutaneous areas affected by radiation-induced toxicity. The device was used in light contact with the tissue surface to ensure consistent energy delivery and minimize beam divergence. A standardized treatment map was followed for each patient, including (1) intra-orally on the tongue, palate and lips; (2) extra-orally on the cheek areas, bilaterally; and (3) bilateral neck. Each site received 1–2 min of irradiation. Patients self-applied the treatment at home twice daily for 9–11 weeks starting on the first day of RT initiation, until 4 weeks following the end of RT. A transparent plastic wrap for single use was used to cover the device to prevent contamination. During the study, patients received standard basic oral care. Figure 1 illustrates the treatment sites to illustrate the protocol and enhance reproducibility.

### Evaluations

At each documentation visit, the following assessment measures were filled: (1) OM and RD grade according to the WHO; (2) The quality of life questionnaire (FHNSI); (3) Subjective satisfaction was graded by the patients using a VAS in which 0 = not satisfied at all and 10 = extremely satisfied. Similarly, ease of use was graded by the patients using a VAS in which 0 = extremely hard to use and 10 = very easy, and comfort of the device was graded from 0 = totally uncomfortable to 10 = very comfortable.

In addition, the patients received a diary in which they recorded the daily treatments. Side effects were recorded throughout the study.

### Statistics

The study sample size is pragmatic and based on the clinically relevant number of patients needed to determine the feasibility of the treatment protocol in this population [34]. The data is summarized using descriptive statistics. Continuous variables are presented as mean and standard deviation or median and interquartile range, and categorical variables are presented as counts or percentages.

According to the protocol, patients were expected to perform 2 daily treatments throughout the RT period. The study hypothesis was that participants will perform at least 50% of the prescribed treatment sessions.

## Results

20 patients were enrolled, 1 patient died before treatment initiation due to disease complication, 1 patient withdrew consent, 1 patient was excluded due to a new onset diabetes diagnosis. 17 patients were analyzed: 5 females (30%) and 12 males (70%), with an average age of 62.4 ± 11.1 (range 43–80 years-old). 8 patients (47%) had smoking history. All patients had squamous cell carcinoma. The most common primary cancer sites were the oral cavity and the nasopharynx. 12 (70%) patients received chemotherapy, with 10 (58%) of them receiving it concurrently with RT (Table 1).

**Table 1:** Demographic and clinical data of enrolled patients

Demographics	NUMBER (%)
**Total enrolled**	20 patients
**Exclusions/withdrawals**	1 patient died before treatment
	1 patient withdrew consent
	1 patient excluded (new diabetes)
**Analyzed patients**	17 patients (100)
**Male**	12 (70)
**Smoking history**	8 patients (47)
**Average age**	62.4 ± 11.1 years (range: 43–80)
**Cancer type**	Squamous cell carcinoma (100%)
**Primary cancer sites**	
Oral cavity:	4 (23.5)
Nasopharynx:	4 (23.5)
Oropharynx:	3 (17.6)
Larynx:	3(17.6)
Skin:	3(17.6)
**Disease stage**	
Stage I:	1 (5.9)
Stage II:	6 (35.3)
Stage III:	7 (41.2)
Stage IVA:	3 (17.6)
**Chemotherapy**	
Total	12 (70.6)
Neoadjuvant	5 (29%)
Concurrent with radiation	10 (58%)
Neoadjuvant + concurrent	3 patients

**Fig. 2:**
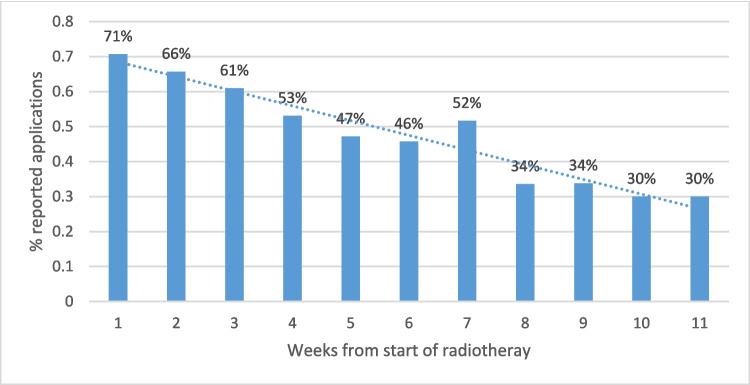
Adherence rate to the treatment schedule outlined in the study protocol, namely applying photobiomodulation treatment, twice daily from the start of RT until 4 weeks after the end of RT, intraorally and to cheek and neck bilaterally

67% of the participants adhered to at least 50% of the recommended PBM sessions to the designated treatment points, twice daily from the start of RT until 4 weeks after the end of RT. In addition, 60% (ranging from 5 to 100%) of the expected treatment sessions were delivered, exceeding the predetermined threshold for compliance. As expected, the compliance rate reduced over time, starting at 71% in the first week and dropping to 30% by week 11 (Fig. 2). Five patients were not included due to missing data (2 screen failure, 1 death, 2 did not fill the diaries).

**Fig. 3:**
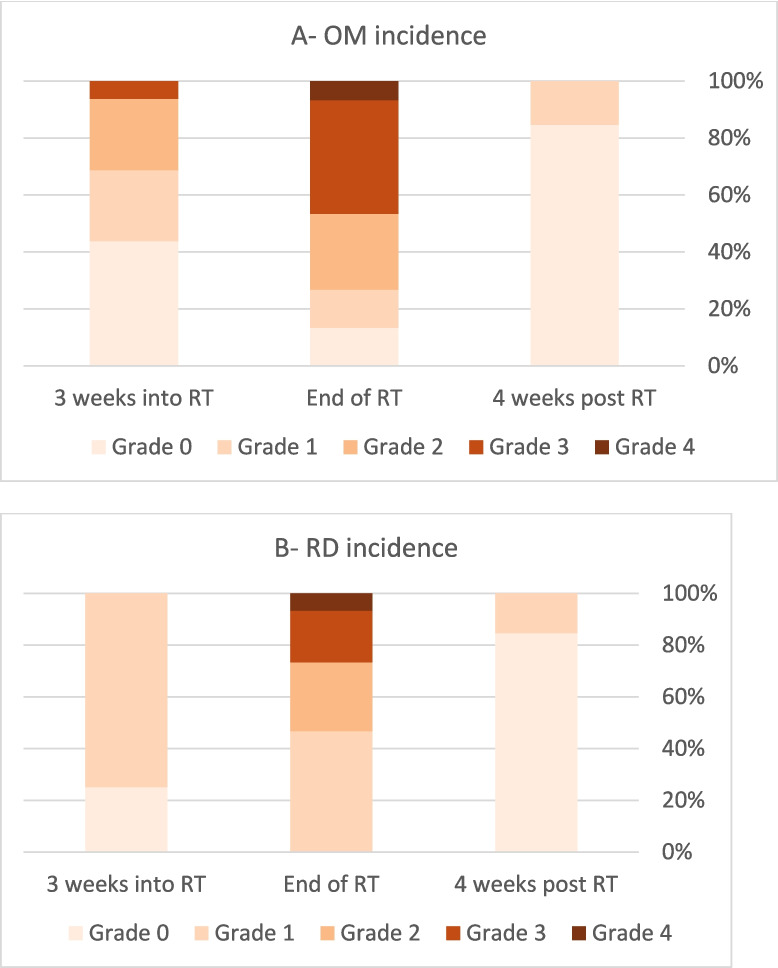
**A** OM incidence at three time points. **B** RD incidence at three time point. OM, oral mucositis; RD, radiation dermatitis

Three weeks after the initiation of RT, one patient missed evaluation. Among the remaining 16 patients, 41.2% (7/16) showed no signs of OM, while 5.9% (1/16) developed grade 3 OM. By the end of RT, 41% experienced grade 3–4 OM (Table 2 and Fig. 3A).

**Table 2:** OM and RD rates at 3 time points. OM- oral mucositis. RD-radiation dermatitis. RT- radiotherapy

Time point	OM GRADE	NO	%	RD GRADE	NO	%
**3 weeks into – RT**	**Grade 0**	7	41.2%	**Grade 0**	4	23.5%
	**Grade 1**	4	23.5%	**Grade 1**	12	70%
	**Grade 2**	4	23.5%			
	**Grade 3**	1	5.9%			
**End of RT**	**Grade 0**	2	11.8%	**Grade 0**	0	0
	**Grade 1**	2	11.8%	**Grade 1**	7	41.2%
	**Grade 2**	4	23.5%	**Grade 2**	4	23.5%
	**Grade 3**	6	35.3%	**Grade 3**	3	17.6%
	**Grade 4**	1	5.9%	**Grade 4**	1	5.9%
**4 weeks post-RT**	**Grade 0**	11	64.7%	**Grade 0**	11	64.7%
	**Grade 1**	0	0	**Grade 1**	2	11.8%
	**Grade 2**	2	11.8%	**Grade 2**	0	0

Regarding RD, no grade 3–4 cases were reported at the three-week mark. By the end of RT one patient (5.9%) developed grade 4 RD. Notably, no severe OM or RD were observed four weeks post treatment (Table 2 and Fig. 3B).

**Fig. 4:**
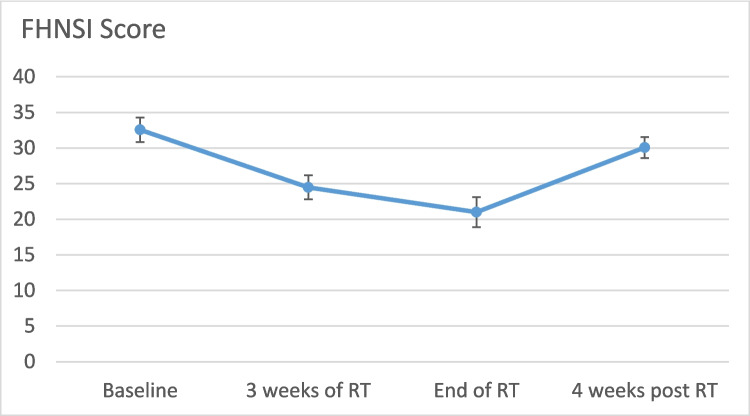
FHNSI score at baseline, 3 weeks after RT, end of RT and 4-weeks post RT. FHNSI- Functional Assessment of Cancer Therapy-Head and Neck Symptom Index, RT- radiotherapy

All patients scored comfort of use as 10 of 10. The average score for ease of use was 9.7 of 10. Three weeks into RT and at its completion, FHNSI scores significantly declined compared to baseline values (32.5 ± 7.6 to 24.4 ± 7.5 and 21 ± 9.4, *p* = 0.001 and *p* < 0.001, respectively). However, by four weeks post-RT, scores returned to near baseline levels (30.07 ± 6.5), with no statistically significant difference observed (Fig. 4). No side effects were reported.

## Discussion

This pilot, prospective, single-arm clinical trial evaluated the feasibility, compliance, and potential efficacy of a self-applied, home-use PBM device for the prevention and treatment of radiation-induced OM and RD in HNC patients undergoing RT with or without concurrent chemotherapy. The study demonstrated that self-applied PBM is feasible, comfortable, and easy to use, with a high level of satisfaction and acceptable patient compliance. The study results will serve to design double blinded RCTs to determine the efficacy of the device in reducing the incidence and severity of OM and RD.

The study obtained a 60% adherence rate to the treatment protocol, which included applying PBM treatments at home to the assigned treatment locations, twice daily from the beginning of RT until four weeks after the end of RT. Over time, compliance decreased as OM and RD worsened, which could have impacted the results. This level of compliance is encouraging, particularly given the challenges associated with self-administered therapies in a home setting, especially in patients that suffer from pain and functional impairment in the applied area. All participants reported high levels of comfort and ease of use indicating that the device was well-tolerated and user-friendly. These findings suggest that home-use PBM devices are a viable option for HNC patients, potentially reducing the burden of frequent clinic visits, which can be particularly challenging for patients undergoing RT, as well as for health care providers.

The incidence and severity of OM observed in this study were comparable with the known literature. At the end of RT, 35.3% of patients developed severe OM (grade 3), and 5.9% developed grade 4 OM. Based on the literature, severe OM occurs in 34%-85% of HNC patients undergoing RT [35–38]. Retrospective data from our department indicate that 36.6% of overall patients developed grade 3 OM, while 31.7% experienced grade 4 OM.

The incidence and severity of RD in this study tended to be better than reported in the literature. At the end of the RT, 17.6% of patients developed severe RD (grade 3), and 5.9% developed grade 4 RD. These rates are better than reported for RD, where severe RD affects 30%-57% of patients [35–38]. According to retrospective data from our department, grade 3 RD occurred in 38.2% of patients, and grade 4 RD in 5.8%. The more pronounced improvement in RD rates compared to OM may be attributed to the flat shape of the applicator, which is better suited for the skin surface, along with the superficial penetration depth of the laser. These findings highlight the possible need for an intra-oral applicator specifically designed to ensure uniform laser distribution within the oral cavity. Notably, only a few cases of severe OM or RD were observed at the end of RT, suggesting that the PBM device may have aided in resolving these conditions over time.

The therapeutic benefits of PBM in mitigating radiation-induced oral mucositis and dermatitis are thought to arise from several complementary biological mechanisms. Absorption of red (620–700 nm) and near-infrared (700–1440 nm) light by endogenous cellular chromophores, most notably mitochondrial cytochrome c oxidase (COX), leads to increased mitochondrial electron transport, enhanced mitochondrial membrane potential, and elevated production of adenosine triphosphate (ATP) [39]. A key mechanistic pathway involves the dissociation of inhibitory nitric oxide from COX, which further augments electron flow and ATP synthesis. This process also generates reactive oxygen species (ROS) and modulates intracellular calcium levels, both of which act as secondary messengers to activate various intracellular signaling cascades [40, 41].These cascades include the activation of transcription factors such as NF-κB, leading to upregulation of genes involved in cell proliferation, migration, differentiation, anti-apoptotic responses, and antioxidant defenses [42]. PBM also promotes angiogenesis primarily by stimulating endothelial cell proliferation, migration, and tube formation through several convergent mechanistic pathways [43]. Additional mechanistic pathways include modulation of light-sensitive ion channels (e.g., TRP channels), which facilitate calcium influx and further influence cellular signaling [41]. The downstream effects of these molecular events are observed at the cellular and systemic levels, including enhanced tissue repair, reduced inflammation, improved wound healing, and neuroprotective effects. Collectively, these mechanisms provide a biologic rationale for PBM’s role in supportive cancer care and underscore the importance of further mechanistic and clinical studies.

Previous research on office-based PBM showed reduced incidence and severity of OM in HNC patients [17, 18, 20, 24]. However, office-based PBM requires frequent clinic visits, which can be logistically and financially burdensome, consume healthcare provider resources, and pose additional challenges for patients undergoing intensive cancer treatment, particularly those who are immunocompromised. The self-applied, home-use PBM device offers a practical and accessible alternative, potentially improving patient utilization of this beneficial therapy. By advancing the conversation from hospital-based to home-based supportive care, this modality offers timely and actionable insights that can directly inform clinical practice and patient-centered care.

The high satisfaction and ease of use reported by patients in this study further support the feasibility of home-based PBM as a complementary approach to managing RT-induced toxicities, while empowering the patient. No side effects were reported.

While the literature is saturated with studies evaluating PBM for the treatment of OM and RD in an office setting, home treatment is rarely reported. One case series reported the use on a home-based device in two patients with OM, three patients with non-healing wounds, and one patient with RD, with effective results [31]. According to another case report, preemptive self-applied PBM showed potential as an effective therapy for lowering OM severity in a HNC patient [44].

To the best of our knowledge, there are no other prospective studies that have evaluated the use of the home device in the setting of OM and RD in HNC patients.

This study has several limitations. First, the small sample size and single-arm design. Second, the lack of randomization and blinding limit the generalizability of the findings. Third, the study did not account for potential confounding factors, such as variations in RT protocols and chemotherapy regimens. Fourth, there was no appropriate control cohort to assess the effect of home-use PBM. Future studies should include a well-defined control group or employ a double-blind sham device design to more accurately evaluate the comparative effectiveness of home-use PBM. Finally, the short follow-up period (four weeks post-RT) may not capture cases of chronic OM [45, 46].

Given the promising feasibility and compliance results, future studies should focus on larger, randomized, placebo-controlled trials to evaluate the efficacy of self-applied PBM in reducing the incidence and severity of OM and RD in HNC patients. These studies should include longer follow-up periods to assess the long-term benefits of PBM and explore potential differences in outcomes based on RT and chemotherapy protocols. Additionally, future research should investigate the cost-effectiveness of home-use PBM devices compared to office-based PBM, as well as the impact of PBM on patient quality of life and treatment adherence.

## Conclusion

In conclusion, this pilot study demonstrates that self-applied, home-use PBM is feasible, comfortable, and well-tolerated by HNC patients undergoing RT. A possible tendency for lower RD incidence was noted. While the study did not provide conclusive evidence of the device's efficacy in reducing OM and RD, the high compliance and satisfaction rates suggest that home-based PBM is a promising approach for managing RT-induced toxicities. Further research is needed to confirm these findings and establish the role of home-use PBM devices in the supportive care of HNC patients.

## Data Availability

The datasets used and/or analyzed during the current study are available from the corresponding author on reasonable request.
